# Genome evolution in major *Escherichia coli *O157:H7 lineages

**DOI:** 10.1186/1471-2164-8-121

**Published:** 2007-05-16

**Authors:** Yongxiang Zhang, Chad Laing, Marina Steele, Kim Ziebell, Roger Johnson, Andrew K Benson, Eduardo Taboada, Victor PJ Gannon

**Affiliations:** 1 Laboratory for Foodborne Zoonoses, Health Canada, Lethbridge, AB, Canada; 2 Laboratory for Foodborne Zoonoses, Health Canada, Guelph, ON, Canada; 3 Department of Food Science and Technology, University of Nebraska, USA

## Abstract

**Background:**

Genetic analysis of *Escherichia coli *O157:H7 strains has shown divergence into two distinct lineages, lineages I and II, that appear to have distinct ecological characteristics, with lineage I strains more commonly associated with human disease. In this study, microarray-based comparative genomic hybridization (CGH) was used to identify genomic differences among 31 *E. coli *O157:H7 strains that belong to various phage types (PTs) and different lineage-specific polymorphism assay (LSPA) types.

**Results:**

A total of 4,084 out of 6,057 ORFs were detected in all *E. coli *O157:H7 strains and 1,751 were variably present or absent. Based on this data, *E. coli *O157:H7 strains were divided into three distinct clusters, which consisted of 15 lineage I (LSPA type 111111), four lineage I/II (designated in this study) (LSPA type 211111) and 12 lineage II strains (LSPA 222222, 222211, 222212, and 222221), respectively. Eleven different genomic regions that were dominant in lineage I strains (present in ≥80% of lineage I and absent from ≥ 92% of lineage II strains) spanned segments containing as few as two and up to 25 ORFs each. These regions were identified within *E. coli *Sakai S-loops # 14, 16, 69, 72, 78, 83, 85, 153 and 286, Sakai phage 10 (S-loops # 91, 92 and 93) and a genomic backbone region. All four lineage I/II strains were of PT 2 and possessed eight of these 11 lineage I-dominant loci. Several differences in virulence-associated loci were noted between lineage I and lineage II strains, including divergence within S-loop 69, which encodes Shiga toxin 2, and absence of the non-LEE encoded effector genes *nleF *and *nleH1-2 *and the *perC *homologue gene *pchD *in lineage II strains.

**Conclusion:**

CGH data suggest the existence of two dominant lineages as well as LSPA type and PT-related subgroups within *E. coli *O157:H7. The genomic composition of these subgroups supports the phylogeny that has been inferred from other methods and further suggests that genomic divergence from an ancestral form and lateral gene transfer have contributed to their evolution. The genomic features identified in this study may contribute to apparent differences in the epidemiology and ecology of strains of different *E. coli *O157:H7 lineages.

## Background

Enterohemorrhagic *E. coli *(EHEC) are associated with gastrointestinal and systemic illness in humans. This illness can range in severity from uncomplicated diarrhea to hemorrhagic colitis and the sometimes fatal hemolytic uremic syndrome [1-3]. EHEC strains possess a number of common virulence traits, such as the production of one or more types of antigenically distinct Shiga toxins (Stx1 and Stx2), a large plasmid that encodes an enterohemolysin, and a chromosomal gene cluster termed the locus of enterocyte effacement (LEE) that is found in most, but not all EHEC serotypes [4,5].

*E. coli *O157:H7 is the EHEC serotype most often associated with disease outbreaks and with the onset of severe disease in the U.S., Canada, Japan, and the U.K. [2,3]. Genomic sequencing of two outbreak-related *E. coli *O157:H7 strains, Sakai and EDL 933, revealed that there are many phage-related sequences and genomic islands scattered throughout the chromosome of this organism and that many of these genetic elements encode potential virulence attributes [6-9]. These *E. coli *O157:H7-specific genomic segments are dispersed throughout 177 different regions of a common genomic backbone that is shared with the distantly related *E. coli *K-12. Known as S-loops and O-islands (OI) in Sakai and EDL933 strains, respectively, some of the regions must be responsible for the virulence characteristics that were acquired during evolution of *E. coli *O157:H7.

*E. coli *O157:H7 strains are believed to comprise a clonal complex of related genotypes that are found worldwide [10]. It has been suggested that *E. coli *O157:H7 arose from the enteropathogenic *E. coli *serotype O55:H7 through sequential acquisition of virulence traits and serotype change [11-13]. A step-wise evolution of *E. coli *O157:H7 from enteropathogenic *E. coli *O55:H7 was recently proposed, based on the properties of specific existent strains that carry intermediate characteristics and are presumed to represent intermediates in the evolution of this EHEC serotype [11,13]. The proposed evolutionary pathway includes lysogenization by an *stx2-*converting phage followed by a shift in serotype from O55 to O157 brought about by acquisition of the O157 *gnd-rfb *locus [14]. The EHEC large plasmid was then acquired by the organism and the ability to ferment sorbitol was lost. The sorbitol-non-fermenting O157:H7 ancestor was subsequently lysogenized with an *stx1*-converting phage and, finally, acquired a frameshift mutation in the *uid*A gene, resulting in loss of β-glucuronidase activity [11]. The validity of this stepwise model is supported by recent comparative genomic hybridization (CGH) studies using *E. coli *O157:H7 whole genome-based oligonucleotide microarrays [13].

It is well recognized that *E. coli *O157:H7 populations have a bovine reservoir and that the organism is likely adapted for life in the ruminant gastrointestinal tract [15-18]. Using Octamer-Based Genome Scanning (OBGS), Kim *et al*., showed that Stx-producing, β-glucuronidase and sorbitol-negative *E. coli *O157:H7 strains have diverged into two distinct lineages, lineages I and II, and that descendants of these two lineages appear to have distinct ecological characteristics [19,20]. Populations of the two lineages are widespread in cattle in both the U.S. and Australia, suggesting that these two lineages have been disseminated throughout the global cattle population [20]. Analysis of a set of nearly 1,500 *E. coli *O157:H7 strains showed that lineage I strains are more commonly associated with human disease than lineage II strains, suggesting that there may be differences in virulence characteristics or transmissibility between these two taxonomic groups of *E. coli *O157:H7 strains [21].

Although high resolution comparative studies have indicated that prophages are associated with divergence of *E. coli *O157:H7 strains [6], systematic analysis of genetic distinctions between lineage I and lineage II strains has only recently been undertaken. We [22] and others [23] have recently reported that the Q anti-terminator gene found upstream of the *stx2 *operon in *E. coli *O157:H7 differs between lineage I and II strains. Possession of the *stx2 *gene is thought to be associated with the occurrence of more severe disease, such as hemolytic uremic syndrome, caused by EHEC strains [24]. In addition, Dowd and Ishizaki [25] recently used oligonucleotide mini-arrays to compare expression of a set of 610 genes between three lineage I and three lineage II strains, noting differential expression of *stx2 *as well as a number of other potentially virulence-associated genes under anaerobic growth conditions. Collectively, these published studies suggest that these lineages are genetically distinct and that lineage-specific genetic differences may be responsible for phenotypic differences between members of these two lineages.

To systematically identify lineage-specific genome segments, microarray-based CGH was used in this study to catalogue genomic alterations that are unique to lineage I or lineage II strains. The oligonucleotide microarray was based on the genome sequences of two lineage I, human outbreak-related *E. coli *O157:H7 strains, Sakai [9] and EDL933 [7] and the nonpathogenic *E. coli *K12 (MG1655) strain [26] and it was used to probe the genomes of a collection of *E. coli *O157:H7 strains. Although significant strain-strain variation was observed, our focus was on genome alterations that were conserved within different strains of a given lineage. Regions of divergence identified by CGH were then cloned and sequenced to gain additional insight into the genomic differences between the two lineages. The results of the study show that many lineage-specific differences in genomic content involve genes that are known or potentially virulence-associated. These findings may be used to identify candidate genes that could confer lineage-specific traits related to unique ecological or virulence characteristics.

## Results

### Validation of microarray data by comparison with sequence data

In the CGH experiments, 6,057 probes from the MWG *E. coli *O157:H7 array set hybridized with a mixture of labelled DNA from the three reference strains (K12, Sakai, and EDL933) yielded adequate signals and these probes were used for all subsequent analysis. For *E. coli *O157:H7 EDL933, 5221/5261 (99.2%) of the probes with 100 % identity to the corresponding sequence gave the expected results (Table 1). Among the 40 probes that were expected to hybridize but did not with DNA from *E. coli *O157:H7 strain EDL933, 13 (0.25%) were negative and 27 (0.5%) were uncertain according to the GACK analysis. While for the *E. coli *O157:H7 Sakai strain only 4951/5335 (93%) of the probes with 100 % identity to the corresponding sequence gave the expected results. For strain Sakai, 39 (0.7%) were negative and 345 (6%) were uncertain based on GACK analysis. However, twenty-one of the probes with 100% identity to *E. coli *Sakai sequence that did not generate a positive signal with *E. coli *Sakai DNA were homologous to ORFs in S-loop#108 [9]. This S-loop is equivalent to OI#57 in *E. coli *O157:H7 EDL933. PCR experiments revealed that the Sakai strain used in this study has a deletion of these ORFs in S-loop#108 while the corresponding OI in EDL933 was intact (data not shown).

### Genomic variability in lineage I and lineage II *E. coli *O157:H7 strains

In order to distinguish lineage-specific differences from strain-strain variability, multiple strains belonging to three different genotypic groups were tested. Our strain set included fifteen different LSPA genotype 111111 strains (lineage I), four different LSPA type 211111 strains (designated lineage I/II in this study) and 12 different lineage II strains of LSPA types 222222, 222221, 222212, and 222211. Characteristics of the strains used in the study are presented in Table 2, and data from microarray hybridization experiments with these *E. coli *O157:H7 strains are presented in the supplemental material [see Additional file 1]. A total of 4,084 of the 6,057 probes hybridized with all *E. coli *O157:H7 strains tested, indicating that this set of genes likely represents the conserved core genome of the ancestral *E. coli *O157:H7 population that has been maintained during its evolution. There were 222 probes that hybridized only with DNA from *E. coli *K12 and not with any of the *E. coli *O157:H7 strains tested, including two probes (ECs1372 and b1894) that were expected to hybridize with EDL933 and Sakai DNA, based on sequence identity. The remaining 1751 probes showed significant variability in microarray hybridization signals among *E. coli *O157:H7 strains (Table 3), and the ORFs that they represent were designated as variably absent or present (VAP).

**Figure 1:**
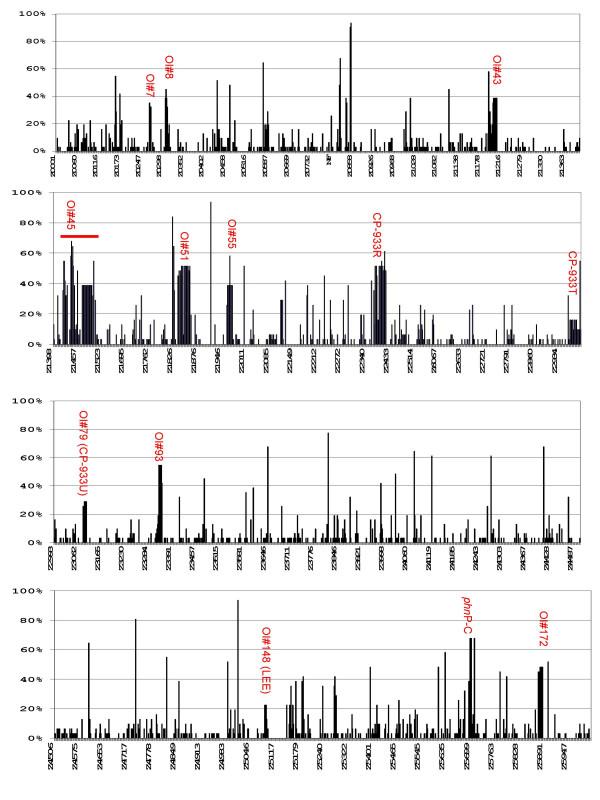
**The distribution of divergent genes among 31 *E. coli* O157:H7 strains as determined in CGH experiments with MWG oligonucleotides**. The position of each ORF in the genome map of *E. coli *O157:H7 strain EDL933 is displayed on the X-axis and the percentage of *E. coli *O157:H7 strains that have divergent ORFs, based on GACK values, is shown on the Y-axis. Specific O islands, prophages and ORFs of interest are identified in the sequence.

**Figure 2:**
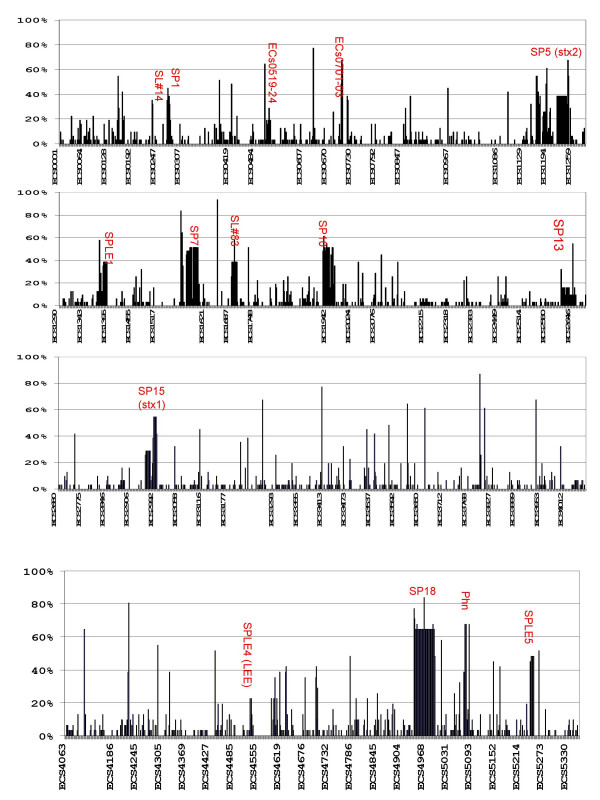
**The distribution of divergent genes among 31 *E. col*i O157:H7 strains as determined in CGH experiments with MWG oligonucleotides**. As in Figure 1 except that the genome map of *E. coli *O157:H7 strain Sakai is used and S-loops and specific ORFs of interest are shown.

Of these 1,751 VAP, 79 hybridized with only one of the 31 *E. coli *O157:H7 strains tested and 662 hybridized with all but one of the 31 *E. coli *O157:H7 strains tested. Initial functional classification of the 1751 VAP genes showed that 506 (29%) were encoded by prophage or phage-like elements found in the K-12, EDL933 and Sakai genomes and 615 (35%) were located within K-island (KI), O-island (OI), or S-loop genomic islands [7,9,26]. The distribution of VAP genes in the genomes of *E. coli *EDL933 and Sakai and the percentage of the 31 *E. coli *O157:H7 strains that were divergent for each gene were plotted (Figures 1 and 2). In this study, "lineage-specific" refers to the presence of single ORFs or ORF clusters exclusively in a given lineage, while "lineage-dominant" refers to the presence of single ORFs or ORF clusters in ≥80% of the strains of one lineage and their absence from ≥90% of strains of other lineages.

### Lineage- and phage type-specific and lineage and phage type-dominant ORFs

**Figure 3:**
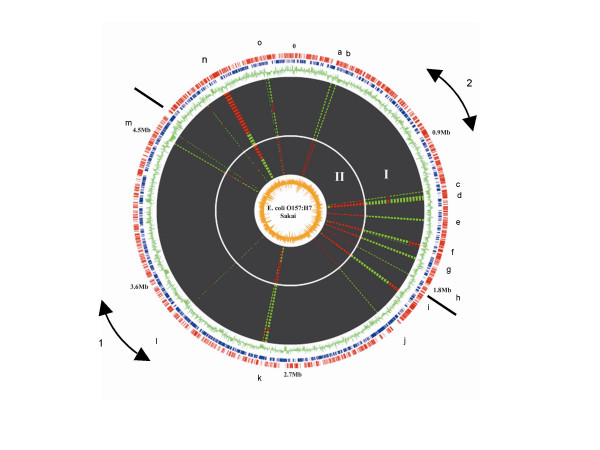
**Relative positions and conservation of lineage-specific and lineage-dominant genome segments in 31 different *E. coli *O157:H7**. Coordinates from the lineage-specific and lineage-dominant ORFs were plotted onto the genome of *E. coli *O157:H7 strain Sakai using the Microbial Genome Viewer [42]. The first ring (outermost) is + strand CDS, the second ring is - strand CDS, and the third ring is Codon Adaptation Index (CAI). Data from the individual strains are given in concentric rings from the periphery to the centre, with lineage I and II strains separated by a solid white ring. Green color indicates that the segment is present and red color indicates its absence. GC content is plotted on the innermost orange ring. a: S-loop#14; b: S-loop#16; c: S-loop#69; d: S-loop#69; r: S-loop#72; f: S-loop#78; g: S-loop#83; h: S-loop#85; i: Sp10 (S-loop#s91, 92, and 93); j: Backbone (ECs2126 - 27); k: S-loop#153; m:S-loop#244 (LEE); n: S-loop#274; o: S-loop#286. The origin and terminus are indicated by black bars extending from the outermost ring. Replichore 1 and replichore 2 are indicated by the outermost numbers.

A total of 132 of the 1,751 VAP ORFs were either specific or dominant to a lineage, LSPA type or PT (Table 4, Figure 3).

#### i) S-loop#14/OI#7

Three lineage I and lineage I/II-specific ORFs, ECs0237, ECs0238, and ECs0239, were identified in S-loop#14/OI#7 by CGH (Table 4). The nucleotide sequence [GenBank:EF112439] of this region in the lineage II strain FRIK 920 was homologous to Sakai sequence, except that a stretch of DNA extending from the 3' end of ECs0237 to the 5' end of ECs0242 was missing. The missing ORFs encode rearrangement hot spot (*rhs*) proteins and hypothetical proteins in *E. coli *Sakai.

#### ii) S-loop#16/OI#8

Eight *E. coli *S-loop#16/OI#8 ORFs were identified as being lineage I and lineage I/II-specific by CGH (Table 4). S-loop#16 corresponds to tandem prophages Sp1 and Sp2 in *E. coli *Sakai, and the majority of lineage I and lineage I/II-specific ORFs in this region were homologous to prophage genes. Repeated attempts to amplify the divergent region in S-loop#16 by long template PCR with FRIK 920 DNA were unsuccessful.

#### iii) S-loop#69/OI#45

S-loop#69/OI#45 corresponds to the *stx2*-converting bacteriophage Sp5, in *E. coli *Sakai. CGH revealed that this region was not only highly divergent but also showed lineage- and LSPA type -dominant patterns of divergence (Table 4). Among the 31 *E. coli *O157:H7 strains examined, only lineage I strain 97701 (PT14) did not have a positive signal for *stx2 *A and B subunit genes. In 97701, other ORFs in this region were also divergent suggesting that bacteriophage Sp5 was not present in its genome.

There were two clusters of lineage and LSPA type divergent ORFs in S-loop#69. The first cluster, consisting of ORFs ECs1160 to ECs1163 located upstream of the *stx2 *genes in *E. coli *Sakai, was missing in all four lineage I/II and the 12 lineage II strains but was conserved in all lineage I strains except strain 97701. The ORFs within this cluster encoded putative bacteriophage proteins and hypothetical proteins.

The second cluster of divergent ORFs in S-loop#69/OI#45 consisted of 21 ORFs, that were missing in 11 out of 12 lineage II strains and present in all four lineage I/II strains and 14 of 15 lineage I strains. These lineage I-dominant ORFs were located downstream of the *stx2 *genes and encoded putative bacteriophage proteins and hypothetical proteins and correspond to the late region of Sp5 of Sakai. PCR primers that flank S-loop#69, were used to amplify the corresponding DNA fragment in the lineage II *E. coli *strain FRIK 920. The nucleotide sequence of the amplicon showed that Sakai Sp5 prophage is not integrated into the chromosome at this site in *E. coli *FRIK 920.

#### iv) S-loop#72/OI#43, 48

S-loop#72 in *E. coli *Sakai, which corresponds to duplicate OI#43 and OI#48 in *E. coli *EDL933, consists of the degenerate prophage SpLE1 in Sakai. S-loop#72 and OI#43,48 are also called tellurite resistance- and adherence-conferring islands because they contain genes responsible for these phenotypes [27]. Putative virulence-associated ORFs located outside of the lineage I and lineage I/II-specific cluster, including the urease genes (ECs1321-ECs1327), genes for tellurite resistance (ECs1351-ECs1358), and *iha *(IrgA homologue adhesin) (ECs1360) [27,28], were found by CGH to be conserved in all *E. coli *O157:H7 strains tested. However, 12 ORFs within S-loop#72 were lineage I and lineage I/II-specific (Table 4). The nucleotide sequence [GenBank:EF112440] of the FRIK 920 amplification product obtained for this region had high similarity to the *E. coli *Sakai sequences, except that a segment 10.8 kb from the 3' end of ECs1377 to the 5' end of ECs1391 was missing. The missing region includes two putative transposases ECs1380 and ECs1381, which were not identified by CGH. With the exception of ECs1382, which encodes a HecB-like protein, and ECs1388 (*pchD*), a PerC-homologue [29], all other lineage I and lineage I/II-specific ORFs in this region encode hypothetical proteins.

#### v) S-loop#78/OI#51

S-loop#78/OI#51, which corresponds to prophage Sp7 in Sakai, contained a cluster of 21 ORFs, located between ECs1574 and ECs1600 of *E. coli *Sakai, that was absent from all lineage II and lineage I/II strains but present in all lineage I strains (Table 4). The S-loop#78 divergent ORFs encoded hypothetical proteins of unknown function and putative bacteriophage-associated proteins. Another ORF present in this region, ECs1588 (*pchE*) [29], which encodes a PerC-homologue, was present in all lineage I and lineage I/II strains but not ten of the twelve lineage II strains tested. Repeated attempts to amplify the divergent region in S-loop#78 by long template PCR with FRIK 920 DNA were unsuccessful.

#### vi) S-loop#83/OI#55

A cluster of 15 lineage I and lineage I/II-specific ORFs, ECs1691-ECs1705, were detected across S-loop#83/OI#55 and its surrounding sequences (Table 4). Five ORFs within the boundaries of S-loop#83, ORFs ECs1693-ECs1697, are homologous to the *prrA-modD-yc73-fepC *gene cluster located on the pyelonephritis and cystitis pathogenicity island of uropathogenic *E. coli *CFT073, which was proposed to be involved in iron uptake in this strain [30]. The S-loop#83 ORFs ECs1698-ECs1699 located immediately downstream from this gene cluster are also conserved in *E. coli *CFT073 and encode putative transport proteins that may be involved in iron transport. Two lineage I- and lineage I/II-specific ORFs located upstream of S-loop#83 encoded hypothetical proteins, and five lineage I- and lineage I/II-specific ORFs located downstream of S-loop#83 encoded components of the *E. coli *phosphotransferase system (PTS), or PTS-dependent dihydroxyacetone kinase enzymes. These are ECs1701 and ECs1702, which together are homologous to the periplasmic trehalase *tre*A in *E. coli *K12 [26], ECs1703, a putative PTS system enzyme I *ycg*C gene, and ECs1704 and ECs1705, which encode putative dihydroxyacetone kinase genes *dhaK1 *and *dhaK2*.

The PCR fragment amplified with DNA from FRIK 920 showed that both the lineage I- and lineage I/II-specific ORFs identified by CGH as well as two putative transposases were missing. The FRIK 920 sequence [GenBank:EF112438] also showed that a portion of *E. coli *K12 DNA sequence (K12 coordinates 1250409–1253544) that was absent from *E. coli *Sakai was present in the chromosome of FRIK 920. The *E. coli *K12 sequence in this region contained portions of b1201 and b1202 ORFs, which encode a PTS-dependent dihydroxyacetone kinase operon regulator *dhaR *and a protein of unknown function with both Pertactin adhesin and autotransporter domains, respectively.

#### vii) S-loop#85/OI#71

Two lineage I- and lineage I/II-specific ORF were detected in S-loop#85/OI#71, which corresponds to Sakai prophage Sp9 (Table 4). The recently described non-LEE encoded effectors *nleA*, *nleH1-2 and nleF *are encoded by ORFs ECs1812, ECs1814 and ECs1815, respectively, within this S-loop [31-33]. Although ECs1814 and ECs1815 were lineage I and lineage I/II-specific, ECs1812 was present in all *E. coli *O157:H7 strains tested. Repeated attempts to amplify this divergent region in S-loop#85 by PCR with FRIK 920 DNA were unsuccessful.

#### viii) Sp10

The Sakai prophage Sp10 region, which is described as a hypervariable locus in EDL933, contains S-loops 91, 92, and 93. Sixteen ORFs within this prophage were observed to be lineage I-specific and one lineage-dominant (Table 4). This region of divergence in lineage I/II and lineage II strains extends from the prophage integrase ECs1929 to the hypothetical protein ECs1955. Most of the downstream ORFs in prophage Sp10, however, are not represented in the MWG microarray, so it could not be determined if these ORFs were present or absent in the *E. coli *O157:H7 strains tested. Based on DNA sequence analysis [GenBank:EF112441], the region corresponding to Sp10 in *E. coli *Sakai DNA was missing entirely from lineage II strain FRIK 920. This prophage contains predominantly ORFs that encode for hypothetical proteins and bacteriophage-associated proteins. Other ORFs of interest within this region include ECs1941 and ECs1942, which encode proteins with low homology to bacteriophage regulatory proteins, and ECs1989, which encodes a putative Cu-Zn superoxide dismutase.

#### ix) S-loop#153/OI#93

S-loop#153/OI#93 corresponds to the *stx1*-converting prophage Sp15 in *E. coli *Sakai. All lineage I/II and lineage II strains and the lineage I PT14 strain LRH6 were divergent in Sakai ORFs ECs2989 to ECs2995 within S-loop#153 (Table 4). The ORFs ECs2989 and ECs2993 encode putative regulatory proteins, while the others encode hypothetical proteins of unknown function or prophage-related proteins. Repeated attempts to amplify this divergent region in S-loop#153 by PCR with FRIK 920 DNA were unsuccessful. However, nucleotide sequence analysis of DNA amplified from the region extending from the flanking region to within the *stx1*-converting prophage showed that integration site of the prophage to be the same in both the lineage II strain FRIK 920 and lineage I strain Sakai (data not shown).

#### x) S-loop#286/OI#172

S-loop#286/OI#172 corresponds to a cryptic prophage-like element SpLE5 in *E. coli *Sakai. Ten ORFs in this region in Sakai and EDL933 were present in all lineage I/II strains and all lineage I strains except those of PT 31 but not lineage II strains (Table 4). The corresponding region [GenBank:EF112443] in *E. coli *FRIK 920 was homologous to *E. coli *Sakai DNA sequence but was missing the DNA segment corresponding to the SpLE5 element (from ECs5242 to ECs5252). The divergent SpLE5 ORFs include bacteriophage-associated genes and several genes encoding hypothetical proteins of unknown function. One of these hypothetical genes, ECs5250, was shown through transposon mutagenesis to be required for intestinal colonization in calves [34]. Another ORF, ECs5252, is a putative transcriptional regulator.

#### xi) KI#71, KI#121, and *E. coli *genomic backbone

Four ORFs in K-island (KI) #71 (b1142, b1147, b1148, and b1152) and four ORFs in KI#121 (b2360, b2361, b2362 and b2363) were identified as being present in all lineage II strains but not lineage I strains. Only the two ORFs in KI#121 (b2360, b2361) were also present in lineage I/II strains. All of these ORFs above encode hypothetical proteins of unknown function (Table 4).

Two ORFs, b1201 and b1202, which were located on the conserved *E. coli *genomic backbone, were only found in *E. coli *K12 and *E. coli *O157:H7 lineage I/II and lineage II strains (Table 4). ORFs b1201 and b1202 encode a putative sensor-type regulator and a putative adhesion and penetration protein, respectively. Lineage I strain *E. coli *Sakai possesses truncated versions of these ORFs.

Two other ORFs found in the *E. coli *genomic backbone, b1519 and b1520, were lineage I and lineage I/II-specific. These ORFs encode a putative trans-aconitate methyltransferase enzyme and a hypothetical membrane protein of unknown function. DNA sequence analysis [GenBank:EF112442] demonstrated that these two ORFs were incomplete in lineage II strain FRIK920.

### Lineage, LSPA type and phage type distribution of ORFs in other virulence-related genomic islands

All genes in S-loop#205/OI#122, which encode two toxins and a PagC-like virulence factor, were conserved among all *E. coli *O157:H7 strains, except for ORF ECs3861. This ORF, which encode a putative adherence factor, was divergent in the lineage II strain FRIK 920. In S-loop#225/OI#138, which contains genes for a fatty acid biosynthesis system, only the putative acyl carrier gene ECs4328 showed variation, and this variation was not lineage-related. All other ORFs in S-loop#225/OI#138 were conserved across all *E. coli *O157:H7 strains examined. In the LEE-containing S-loop#244/OI#148 [5,31], all ORFs were conserved across all *E. coli *O157:H7 strains examined, except for five ORFs encoding hypothetical proteins within the prophage SpLE4 region of the LEE (ECs4534, ECs4535, ECs4537, ECs4542, and ECs4544), which encode a putative integrase and genes for hypothetical proteins of unknown function. These ORFs were missing in seven lineage II strains (LRH13, R1797, EC2000623, EC20000703, EC20020119, FRIK1985, and EC970520). The conserved LEE genes included *eae*, which encodes γ-intimin, *tir *(translocated intimin receptor), *map *(mitochondrial-associated protein), the genes for the type III secretion system (*escCDFJRSTUV*, *cesDT*, and *sepDLQZ*), and genes encoding the system's other secreted proteins (*espA*, *espB*, *espD*, and *espF*) [35]. Homologues of non-LEE encoded effector *nleABCD *genes of *Citrobacter rodentium *(ECs1812, ECs3857, ECs0847, and ECs0850) [31,33,36], were present in all *E. coli *O157:H7 strains tested, although non-LEE encoded effectors *nleH1-2 *(ECs1814) and *nleF *(ECs1815) of S-loop#85 [31,32] and putative transcriptional regulator *pchD *(ECs1388) of S-loop#72 [29], were both lineage I and lineage I/II-specific (see above). Other virulence-associated genes, such as *espF *(ECs2715), enterotoxin-encoding *sen *(ECs3855), porcine EPEC O45 *paa *gene homologue (ECs1772), and calcium-binding and heat-extractable autotransporter gene *cah *(ECs1396), were present in all of the *E. coli *O157:H7 strains examined [37-41].

### Genomic characteristics of the lineage-specific segments

To visualize the distribution of the lineage-specific and lineage-dominant genome segments, the segments were mapped onto the O157:H7 strain Sakai genome using Microbial Genome Viewer [42] along with plots of Codon Adaptation Indices (CAI) and GC content. As shown in Figure 3, the lineage-specific segments (denoted by letters a-o) are distributed throughout several segments of the genome, with two main clusters (cluster c-j and cluster m-b). Correlation exists between positioning of the lineage-specific segments and the origin and terminus of chromosome replication. Nearly half of the lineage I-specific segments (c-j) are concentrated in a 1.0 Mb segment of the genome that includes the replication terminus (at position 1.9 Mb) while several segments in the m-n region are likewise clustered near the replication origin, positioned at 4.8 Mb. However, there appears to be some bias in the distribution of the lineage-specific segments with respect to the two replichores, with segments n-h occurring within replichore 2 while only segments i-m are found in replichore 1. Replichore 1 is 290 Kb longer than replichore 2 in the Sakai genome sequence [9], which is a lineage I strain, and it is possible that symmetry is a driving force in shaping the genomes of the lineage II strains.

### Genomotyping of *E. coli *O157:H7 strains

**Figure 4:**
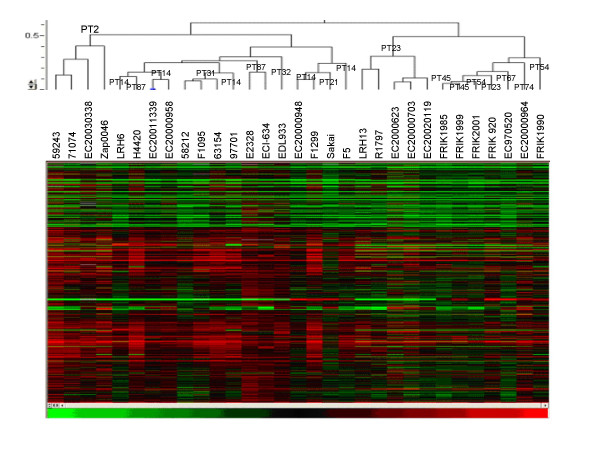
**The dendrogram generated by CGH analysis of *E. coli *O157:H7 strains**. Data generated from two arrays for each strain have been averaged and analyzed with acuity 3.1. The 6057 locus tags of open reading frames of three *E. coli *strains (EDL933, Sakai, and K12 MG1655) in the image were organized alphabetically. The dendrogram was generated by hierarchical cluster analysis (Pearson correlation) of log2 (RAT2N) of each strain with Acuity 3.1. In the colour scheme at the bottom of above figure, the brightest green corresponds to spots that are absent (divergent) with high certainty, the brightest red indicates spots that are present (conserved) with the greatest certainty, the black indicates spots that are uncertain or slightly divergent.

A dendrogram based on the Pearson correlation analysis of microarray data for all 31 *E. coli *O157:H7 strains is presented in Figure 4. Three distinct groups of strains, corresponding to lineage I, lineage I/II (LSPA 211111/PT2) and lineage II (LSPA types 222222, 222221, 222211, and 222212) strains are evident. The three PT31 strains within lineage I are grouped close to each other in the dendrogram. In contrast, PT14 and PT23 strains (the predominant strains in lineage I and lineage II groups, respectively) display variable genetic distances with respect to other strains of the same PT and other strains of different PTs within the same OBGS lineage.

## Discussion

To our knowledge this is the first time that CGH has been applied to such a diverse collection of *E. coli *O157:H7 strains. Results of this study suggest that CGH is a robust and discriminating method for comparing large numbers of *E. coli *O157:H7 strains. Twenty-one of the oligonucleotides with divergent signals for Sakai DNA formed part of the S-loop#108/OI#57 genomic island which was confirmed to be missing from both of the Sakai strains in our collection (data not shown), suggesting that this element was lost during storage or subculture in the laboratory. There are a number of phage-related ORFs in S-loop#108 (Sp12), and the contribution of this gene cluster, if any to the survival and/or virulence of *E. coli *O157:H7 strains in nature is unknown. The high level of agreement between the CGH hybridization signals observed and the expected results for ORFs that were reported to be present and those that were reported to be divergent suggests that CGH is a reliable method of determining genomic composition. Furthermore, all areas of lineage I-specific divergence were in clusters of two or more ORFs, so it seems unlikely that the divergent regions observed in this study are a result of printing or hybridization errors.

An additional line of evidence in support of the reliability of the CGH data was obtained from parallel selective subtractive hybridization (SSH) studies conducted by our research group [43]. CGH identified all of the nine lineage I-dominant chromosomal genomic regions identified by SSH as well as two additional ones. Finally, in several of the areas where lineage I-specific divergence was inferred using CGH, sequence differences between these two lineages were verified following amplification, cloning and sequencing of DNA from the lineage II *E. coli *strain FRIK 920. Taken together, these findings represent a rigorous and comprehensive validation of the CGH data presented in this study.

The CGH data presented in this study provide evidence of both regions of genomic stability and regions of genomic variability that exist within populations of *E. coli *O157:H7. A total of 4084 oligonucleotides hybridized with DNA from all of the 31 *E. coli *O157:H7 strains tested. The ORFs which hybridized with these oligonucleotide probes approximate the conserved portion of the *E. coli *O157:H7 genome and likely include the core genes required for survival of this bacterial pathogen [44,45]. Wick *et al *identified 4230 conserved genes in *E. coli *O157:H7(:H-) and closely related *E. coli *O55:H7 strains using the same oligonucleotide set that was used in this study [13], however, a very limited number of O157 and O157-related strains were examined in the latter study. In contrast, Dobrindt *et al*. identified 3100 core genes in the genomes of 26 *E. coli *strains of different serotypes associated with both intestinal and extra-intestinal illness [44]. It would seem reasonable to hypothesize that the larger the collection of strains from a specific *E. coli *serotype and the more diverse the collection of *E. coli *strains, the fewer core genes and the more VAP genes that would be encountered.

A total of 1751 VAP ORFs were identified in *E. coli *O157:H7 strains in the present study. These ORFs existed as clusters across the *E. coli *Sakai and EDL933 chromosomes (Figures 1 and 2). The number of VAP ORFs identified in this study was significantly higher than that identified in the CGH study by Ogura *et al*. [46] which employed an *E. coli *Sakai-based microarray to examine genomic variability within eight human *E. coli *O157:H7 isolates. The higher number of VAP ORFs observed in this study, likely reflects the higher number of strains tested, the inclusion of both human and bovine-derived strains, and the fact that the MWG microarray used in this study is based on ORFs from *E. coli *O157:H7 Sakai and EDL933 strains and an *E. coli *K12 strain rather than just *E. coli *Sakai.

In addition to genomotyping an extensive collection of *E. coli *O157:H7 strains, we used CGH to compare the genomic profiles of *E. coli *O157:H7 lineage I, lineage I/II and lineage II strains. These observations extend well beyond those obtained from previous *E. coli *K12 and O157:H7 microarray studies [13,44-46] and other genome analysis techniques such as WGPCR Scanning [47]. CGH shows promise as a method that can not only verify the existence of the lineages and categorize strains into subtypes, but also of providing insight into specific genetic differences that could be related to differences in their ecology and evolution. The 132 VAP ORFs that were differentially distributed between the two OBGS lineages were of particular interest in this study, since these ORFs likely confer to strains possessing them the unique ecological or virulence characteristics associated with these lineages.

These 132 lineage-divergent VAP ORFs were clustered within several chromosomal regions, many of which were associated within *E. coli *O157:H7 Sakai and EDL933 S-loops and OIs.

Prophage were originally suspected as either causing or at least correlating with divergence of the genomes in lineage I and lineage II strains [19] and other studies have shown that prophage regions of the genome are highly polymorphic in different O157:H7 strains [47-51]. It is therefore not surprising that many of the VAPs occur within prophage segments because these elements have significant homology to other prophages in the genome, making them hotspots for recombination. Moreover, these regions of the genome also are topologically distinct. The results of distribution mapping of the lineage-specific VAPs (Figure 2) also implies bias, suggesting that genome symmetry may be superimposed onto homology and topology as driving forces shaping the genome during divergence of the two lineages. When combined with the fact that some of the genes present in the affected prophage or pathogenicity islands could influence expression of virulence genes (e.g. the perC-like genes ECs1388 and ECs1588), it seems reasonable to conclude that multiple evolutionary forces may be working on these genome segments.

For many of these divergent regions, a bias in PT was also evident. Divergent ORFs identified in S-loops 69, 78, and 153 and in Sp10 (S-loops 91, 92 and 93) were absent in all lineage II strains. Similarly, lineage I PT31 strains and lineage II strains also lacked the same ORFs in S-loop#286. Lineage I/II strains were all of the same LSPA type, 211111, and of PT 2 and formed a distinct cluster in a dendrogram based on the Pearson correlation analysis of microarray data for all 31 *E. coli *O157:H7 strains (Figure 4). PT23 was the most common PT of lineage II strains included in this study. All PT23 strains from Canada grouped together within the lineage II cluster on this dendrogram, despite different isolation dates, host origins, and geographic origin within Canada. In contrast to the apparent clonality of PT2, PT31, and PT23 strains, strains of PT14, the predominant lineage I PT, were highly divergent on this dendrogram and some PT14 strains appeared to be closely related to other PTs, such as PT87, PT21, and PT31. These results suggest that there is a relationship between genomotype and lineage and between genomotype and some PTs for *E. coli *O157:H7 strains, however, a larger number of strains from different geographical regions need to be examined to verify these relationships.

The lineage, LSPA type and PT-specific and lineage, LSPA type or PT-dominant VAP ORFs identified in this study were associated with several known or suspected virulence genes, including regions of divergence within S-loop 69, which encodes Stx2. A number of studies have shown divergence in the structure of prophages encoding Stx genes in *E. coli *O157:H7, their insertion sites within the genome, and the type and level of toxin produced [22,47,50,52,53]. In addition to this, the absence of the non-LEE encoded effector genes *nleH1-2 and nleF *and the *perC *homologue gene *pchD *in lineage II strains are likely to affect their phenotype. However, further study is required to determine the precise role of these differences in the genomes *E. coli *O157:H7 lineage I and lineage II strains on their ecology and virulence.

## Conclusion

CGH was used to evaluate genomic variability within a collection of 31 *E. coli *O157:H7 strains. A total of 4084 ORFs were detected in all of the strains, suggesting that they represent core genes conserved in all *E. coli *O157:H7 strains. Among the 1751 VAP ORFs were clusters of ORFs associated with bacteriophages and other genetically mobile elements. Several clusters of ORFs were lineage-specific or dominant. A number of the genes within lineage-specific or dominant ORF clusters have been associated with virulence in *E. coli *O157:H7. Nucleotide sequencing of lineage-specific or dominant regions in the lineage II *E. coli *FRIK 920 confirmed that the alterations in the genome detected by CGH are the results of genomic deletions or insertions. The genomic composition of these strains within lineages and subgroups suggests that both genomic divergence from an ancestral form and the lateral transfer of gene clusters have contributed to their evolution.

## Methods

### Bacterial strains and preparation of genomic DNA

The 15 lineage I (including EDL933 and Sakai strains for which the genomic sequence has been determined) (LSPA 111111), four PT 2 (LSPA 211111) strains and 12 lineage II (LSPA 222222) *E. coli *O157:H7 strains included in this study were obtained from a variety of human and bovine sources (Table 2). OBGS types of all 31 strains were determined as previously described [19]. Phage typing of *E. coli *O157:H7 isolates was carried out as described previously [54]. The *E. coli *strains were grown overnight in 45 mL of Brain-Heart-Infusion (BHI) broth. The cultures were centrifuged at 8000 rpm for 10 minutes and the pellet was dissolved in 15 mL of 10 mM NaCl, 20 mM Tris-HCl (pH 8.0), 1 mM EDTA, 100 μg/mL proteinase K and 0.5% SDS. This suspension was incubated at 50°C for 2 h and extracted with an equal volume of phenol:chloroform:isoamyl alcohol (25:24:1). Following centrifugation for 10 min at 8000 rpm, the upper phase was removed and precipitated by adding 0.1 volume of 3 M NaOAc (pH 5.2) and 2 volumes of 99% ethanol. The DNA precipitate was then spooled out of the solution using a sterile glass rod, washed with 70% ethanol, and dissolved in 5 mL of TE (10 mM Tris-HCl, 1 mM EDTA, pH 8.0) buffer.

### Array preparation

Corning Ultra-Gap II slides (Corning, Acton, MA) were spotted with the MWG *E. coli *O157:H7 array set (MWG Biotech). The MWG array consists of 6167 50-mer oligonucleotides covering the genomes of *E. coli *K-12 (MG1655) [26] and *E. coli *O157:H7 strains Sakai (RIMD 0509952) [9] and EDL933 (ATCC700927) [7]. Prior to use, each array was pre-hybridized at 50°C in a solution of 5× SSC, 0.1% SDS and 0.1% BSA for one h, washed completely in dH_2_O, rinsed with isopropanol, and then centrifuged and dried.

### Hybridizations

5 μg of test genomic DNA was digested with *Eco*RV and *Pst*I restriction enzymes, 3 μg of which was labelled with ULYSIS Alexa Fluor 647 dye (Invitrogen, Burlington, ON.). The laboratory strain *E. coli *K-12 (MG1655) and the two sequenced O157:H7 strains, Sakai (RIMD 0509952) and EDL933 (ATCC700927), were digested in an analogous fashion, and 1 μg of each was combined and labelled with Alexa Fluor 546 to create the reference DNA (Invitrogen, Burlington, ON). Unincorporated dye was removed using Qiaquick PCR purification kit (Qiagen, Mississauga, ON), according to the manufacturer's instructions, and the DNA eluted in 30 μl of TE (10/1) buffer. Labelled DNA was vacuum-dried and resuspended in 20 μl dH_2_O. A 70 μl hybridization solution consisting of 30% formamide, 5× SSC, 0.1% SDS, 0.1 mg/ml sonicated salmon sperm DNA, and equal amounts of test and reference DNA, each containing at least 30 pmol of incorporated dye, was denatured at 95°C for 5 min and briefly centrifuged to collect the contents. DNAs were hybridized to the array overnight (~16 hours) at 42°C and washed according to the modified Corning method (Corning). Arrays were scanned with a GenePix 4000B scanner (Axon Instruments, Redwood City, CA) and processed using GenePix Pro 5.0. Two slides were hybridized per strain with two dye-swap repeats per slide.

### Microarray data analysis

Microarray data were normalized using the Ratio-based and Lowess method in Acuity 3.1 (Axon instruments) before analysis. The normalized data for all 31 strains were converted to log_2 _(Fluor 647/Fluor 546) in Acuity 3.1 and subsequently analyzed in Microsoft Excel. Control, blank, and test spots with a mean intensity below that of the mean of all negative controls were removed from the analysis. The arithmetic mean of the remaining spots across the four duplicates was taken to construct the dataset. Acuity 3.1 was then used to construct a Pearson centred hierarchical dendrogram and GACK (Genomotyping Analysis by Charles Kim) [55] was used to generate a cut off value for identifying the presence or absence of genes.

### Identification of lineage- and phage type-dominant ORF clusters

The presence or absence of each ORF was compared for all 15 lineage I, 4 lineage I/II and 12 lineage II *E. coli *O157:H7 strains included in the study, and ORFs that were variably absent and present (VAP) within this strain collection were identified. The proportion of strains of each OBGS lineage and phage type (PT) containing each VAP ORF was determined. ORFs that were present in 100% of strains in a lineage or PT and absent in all strains of the other lineage or PTs were designated as lineage or PT-specific. ORFs that were present in greater than 80 % of strains in a lineage or PT and absent in greater than 92 % of strains of the other lineage or PTs were defined as lineage or PT-dominant.

For visualization of VAP distribution, each locus tag was given a binary score, 1 for presence and 0 for absence based on GACK analysis. A single composite table was generated for all strains and sorted in Microsoft Excel to identify lineage-specific polymorphisms. This generated a set of 132 loci showing a lineage-specific distribution. The binary distribution was then represented in RGB format (1 = green, 0 = red), converted to a single text file, and the file uploaded and represented on the Microbial Genome Viewer [42].

DNA sequences from the lineage-conserved regions flanking lineage and PT-dominant regions were used to design primers for PCR amplification, cloning and DNA sequence analysis of these divergent regions (Table 5). DNA from the lineage II strain *E. coli *FRIK 920 was used to amplify divergent regions corresponding to *E. coli *Sakai S-loops 14, 16, 69, 72, 78, 83, 153, and 286, backbone regions b1519-b1520 and SP10. These divergent regions were amplified by PCR using AmpliTaq^® ^polymerase (Applied Biosystems, Foster City, CA) or long template PCR using the Advantage™ 2 PCR Kit (BD Biosciences Clontech, Palo Alto, CA) and then cloned into the pGEM-T Easy vector (Promega, Madison, WI), the pCR 2.1 TOPO TA vector (Invitrogen, Carlsbad, CA), or the Expand cloning kit (Roche Applied Science). The primers and annealing temperatures used in these reactions are summarized in Table 5. The cloned sequences were analyzed on an ABI Prism 277 DNA sequencer (Applied Biosystems, Foster City, CA) by primer walking, using purified cosmid or plasmid DNA as template, and the sequences obtained were deposited in GenBank [GenBank:EF112438, EF112439, EF112440, EF112441, EF112442 and EF112443]. The NCBI BLASTN program was used to identify differences between *E. coli *FRIK 920 sequences and *E. coli *Sakai [GenBank:BA000007] sequences.

## Abbreviations

CGH: comparative genomic hybridization; LEE: locus for attachment and enterocyte effacement; OI: O-island; PAI: pathogenicity island; PT: phage type; Sp: Sakai prophage; SPLE – Sakai prophage-like element; TAI: tellurite resistance- and adherence-conferring island, VAP: variably present or absent

## Authors' contributions

YZ planned and carried out experimental work, data analysis and writing of the manuscript. CL carried out experimental work and complied and analyzed microarray data. MS planned experimental work, assisted with sequence data analysis, and revision of the manuscript. KZ planned experimental work and provided bacterial strains. RJ assisted with project planning and experimental design and revision of the manuscript. CC planned experimental work and was responsible for microarray fabrication. AKB helped plan experimental work, provided bacterial strains, assisted with data analysis and revision of the manuscript. ET assisted with data analysis and revision of the manuscript. VJG planned experimental work, helped with data analysis and preparation of the manuscript. All authors have read and approved the final manuscript.

## Supplementary Material

Additional File 1 *E. coli *O157:H7 microarray dataset Click here for file

## Figures and Tables

**Table 1:** Summary of BLASTN results of MWG oligonucleotide probes queried against genomes of *E. coli *O157:H7 strains EDL933, and Sakai, and K-12 strain MG1655

			ORFs with 100% identity to					Less than 100% identity to	Total probes
Target	K12	EDL933	Sakai	EDL933 and Sakai	EDL933 and K12	Sakai and K12	K12, EDL933 and Sakai	K12, EDL933, or Sakai	
Probe No.	4269	5261	5335	5232	3659	3655	3654	84	6057

**Table 2:** *E. coli *O157:H7 strains used in CGH experiments.

Name	Serotype	Phage type	Source	LSPA type	*stx1*	*stx2*	
97701	O157:H7	14	Human	111111	+	-	
LRH6	O157:H7	14	Human	111111	-	+	
EC20011339	O157:H7	14	Bovine	111111	+	+	
F1299	O157:H7	14	Bovine	111111	+	+	
F5	O157:H7	14	Bovine	111111	+	+	
63154	O157:H7	31	Human	111111	+	+	
58212	O157:H7	31	Human	111111	+	+	
F1095	O157:H7	31	Bovine	111111	+	+	
H4420	O157:H7	87	Bovine	111111	+	+	
E2328	O157:H7	87	Bovine	111111	+	+	
ECI-634	O157:H7	87	Bovine	111111	+	+	
Sakai	O157:H7	32	Human	111111	+	+	
EDL933	O157:H7	21	Human	111111	+	+	
EC20000948	O157:H7	14	Human	111111	+	+	
EC20000958	O157:H7	14	Human	111111	+	+	
59243	O157:H7	2	Human	211111	-	+	
71074	O157:H7	2	Human	211111	-	+	
EC20030338	O157:H7	2	Human	211111	-	+	
Zap0046	O157:H7	2	Human	211111	-	+	
EC970520	O157:H7	67	Bovine	222222	+	+	
LRH13	O157:H7	23	Human	222222	+	+	
R1797	O157:H7	23	Human	222222	+	+	
EC20020119	O157:H7	23	Bovine	222222	+	+	
EC2000623	O157:H7	23	Bovine	222222	+	+	
EC20000703	O157:H7	23	Bovine	222222	+	+	
FRIK 920	O157:H7	23	Bovine	222222	+	+	
FRIK1999	O157:H7	23	Bovine	222222	+	+	
FRIK1985	O157:H7	45	Bovine	222221	+	+	
FRIK1990	O157:H7	54	Bovine	222222	+	+	
FRIK2001	O157:H7	54	Bovine	222211	-	+	
EC20000964	O157:H7	74	Human	222212	-	+	

**Table 3:** Genome ORF variability in 15 lineage I, four lineage I/II and 12 lineage II *E. coli *O157:H7 strains

Divergent^1^ Strain # Group	ORFs	Phage-related genes	Phage-unrelated genes	OI, KI, SL^2^-related genes	OI, KI, SL^2^-unrelated genes (backbone)
0	4084	689	3395	1077	3007
1	662	78	584	95	567
2	190	33	157	43	147
3–6	291	79	212	90	201
7–12	161	79	82	91	70
13–18	141	98	43	97	44
19–25	136	87	49	97	39
26–29	91	30	61	52	39
30	79	22	57	50^3^	29
31	222	84	138	190	32
Total	1751	506	1245	615	1136

^1^Indicates the number of strains lacking specific ORFs. ^2^OI = O-island, KI = K-island, SL = S-loop. ^3^These ORFs are only from K-islands.

**Table 4:** Distribution of VAP ORFs identified in the microarray study

Location	ORF	Function	Lineage I-LSPA 111111	PT2-LSPA 211111	Lineage II-LSPA 222222
			n = 15	n = 4	n = 12
S-loop#14/OI#7	ECs0237 (Z0268)	RhsG core protein with extension	100%	100%	0%
S-loop#14/OI#7	ECs0238 (Z0269)	hypothetical protein	100%	100%	0%
S-loop#14	ECs0239	hypothetical protein	100%	100%	0%
S-loop#16	ECs0272	putative transcription antitermination protein	100%	100%	0%
S-loop#16/OI#8	ECs0273 (Z0308)	hypothetical protein	100%	100%	0%
S-loop#16/OI#8	ECs0274 (Z0309)	repressor protein CI	100%	100%	0%
S-loop#16	ECs0275	Cro repressor	100%	100%	0%
S-loop#16/OI#8	ECs0276 (Z0310)	regulatory protein cII	100%	100%	0%
S-loop#16/OI#8	ECs0277 (Z0311)	replication protein	100%	100%	0%
S-loop#16/OI#8	ECs0279 (Z0313)	replication protein	100%	100%	0%
S-loop#16/OI#8	ECs0280 (Z0317)	putative tail fiber protein	100%	100%	0%
S-loop#69/OI#45	ECs1160 (Z1424)^1^	putative integrase	93%	0%	0%
S-loop#69/OI#45	ECs1161 (Z1425)^1^	putative excisionase	93%	0%	0%
S-loop#69/OI#45	ECs1162 (Z1426)^1^	hypothetical protein	93%	0%	0%
S-loop#69/OI#45	ECs1163 (Z1428)^1^	hypothetical protein	93%	0%	0%
S-loop#69/OI#45	ECs1219 (Z1475)^2^	putative small subunit terminase	93%	100%	8%
S-loop#69/OI#45	ECs1220 (Z1476)^2^	putative terminase large subunit	93%	100%	8%
S-loop#69/OI#45	ECs1221 (Z1477)^2^	putative portal protein	93%	100%	8%
S-loop#69/OI#45	ECs1222 (Z1478)^2^	hypothetical protein	93%	100%	8%
S-loop#69/OI#45	ECs1223 (Z1479)^2^	hypothetical protein	93%	100%	8%
S-loop#69/OI#45	ECs1224 (Z1480)^2^	hypothetical protein	93%	100%	8%
S-loop#69/OI#45	ECs1225 (Z1481)^2^	hypothetical protein	93%	100%	8%
S-loop#69	ECs1226 ^2^	hypothetical protein	93%	100%	8%
S-loop#69/OI#45	ECs1227 (Z1482)^2^	hypothetical protein	93%	100%	8%
S-loop#69/OI#45	ECs1228 (Z1483)^2^	putative tail fiber protein	93%	100%	8%
S-loop#69/OI#45	ECs1232 (Z1486)^2^	hypothetical protein	93%	100%	8%
S-loop#69/OI#45	ECs1233 (Z1487)^2^	putative tail tip fiber protein	93%	100%	8%
S-loop#69	ECs1234 ^2^	putative outer membrane protein	93%	100%	8%
S-loop#69/OI#45	ECs1235 (Z1488)^2^	hypothetical protein	93%	100%	8%
S-loop#69/OI#45	ECs1236 (Z1489)^2^	putative outer membrane precursor	93%	100%	8%
S-loop#69/OI#45	ECs1237 (Z1490)^2^	hypothetical protein	93%	100%	8%
S-loop#69/OI#45	ECs1238 (Z1491)^2^	hypothetical protein	93%	100%	8%
S-loop#69/OI#45	ECs1239 (Z1492)^2^	hypothetical protein	93%	100%	8%
S-loop#69/OI#45	ECs1240 (Z1493)^2^	hypothetical protein	93%	100%	8%
S-loop#69/OI#45	ECs1241 (Z1494)^2^	hypothetical protein	93%	100%	8%
S-loop#69/OI#45	ECs1242 (Z1495)^2^	hypothetical protein	93%	100%	8%
S-loop#72/OI#43,48	ECs1377 (Z1635)	hypothetical protein	100%	100%	0%
S-loop#72/OI#43,48	ECs1378 (Z1636)	hypothetical protein	100%	100%	0%
S-loop#72/OI#43,48	ECs1379 (Z1637)	hypothetical protein	100%	100%	0%
S-loop#72/OI#43,48	ECs1382 (Z1640)	HecB-like protein	100%	100%	0%
S-loop#72/OI#43,48	ECs1383 (Z1641)	hypothetical protein	100%	100%	0%
S-loop#72/OI#43,48	ECs1384 (Z1642)	hypothetical protein	100%	100%	0%
S-loop#72/OI#43,48	ECs1385	hypothetical protein	100%	100%	0%
S-loop#72/OI#43,48	ECs1386 (Z1643)	hypothetical protein	100%	100%	0%
S-loop#72/OI#43,48	ECs1387 (Z1644)	hypothetical protein	100%	100%	0%
S-loop#72/OI#43,48	ECs1388	putative transcriptional regulator, *pch*D	100%	100%	0%
S-loop#72/OI#43,48	ECs1389 (Z1645)	hypothetical protein	100%	100%	0%
S-loop#72/OI#43,48	ECs1390 (Z1646)	hypothetical protein	100%	100%	0%
S-loop#78/OI#51	ECs1574 (Z1836)	putative integrase	100%	0%	0%
S-loop#78	ECs1576 (Z1839)	hypothetical protein	100%	0%	0%
S-loop#78/OI#51	ECs1578 (Z1840)	hypothetical protein	100%	0%	0%
S-loop#78	ECs1579	hypothetical protein	100%	0%	0%
S-loop#78/OI#51	ECs1580 (Z1841)	hypothetical protein	100%	0%	0%
S-loop#78	ECs1581	hypothetical protein	100%	0%	0%
S-loop#78/OI#51	ECs1582 (Z1842)	hypothetical protein	100%	0%	0%
S-loop#78	ECs1583	hypothetical protein	100%	0%	0%
S-loop#78/OI#51	ECs1584 (Z1843)	hypothetical protein	100%	0%	0%
S-loop#78	ECs1585	hypothetical protein	100%	0%	0%
S-loop#78/OI#51	ECs1590 (Z1847)	putative major head protein	100%	0%	0%
S-loop#78/OI#51	ECs1591 (Z1848)	putative prohead protease	100%	0%	0%
S-loop#78/OI#51	ECs1592 (Z1849)	putative head portal protein	100%	0%	0%
S-loop#78/OI#51	ECs1593 (Z1850)	putative head-tail adaptor	100%	0%	0%
S-loop#78/OI#51	ECs1594 (Z1851)	hypothetical protein	100%	0%	0%
S-loop#78/OI#51	ECs1595 (Z1852)	hypothetical protein	100%	0%	0%
S-loop#78	ECs1596	hypothetical protein	100%	0%	0%
S-loop#78/OI#51	ECs1597 (Z1853)	putative terminase small subunit	100%	0%	0%
S-loop#78/OI#51	ECs1598 (Z1854)	putative terminase large subunit	100%	0%	0%
S-loop#78/OI#51	ECs1599 (Z1856)	hypothetical protein	100%	0%	0%
S-loop#78/OI#51	ECs1600 (Z1857)	hypothetical protein	100%	0%	0%
S-loop#83/OI#55 upstream region	ECs1691 (Z1959)	orf, hypothetical protein; ycgR	100%	100%	0%
S-loop#83/OI#55 upstream region	ECs1692 (Z1960)	orf, hypothetical protein; ymgE	100%	100%	0%
S-loop#83/OI#55	ECs1693 (Z1961)	putative TonB dependent outer membrane receptor	100%	100%	0%
S-loop#83/OI#55	ECs1694 (Z1962)	molybdenum transport protein	100%	100%	0%
S-loop#83	ECs1695	hypothetical protein	100%	100%	0%
S-loop#83/OI#55	ECs1696 (Z1963)	hypothetical protein	100%	100%	0%
S-loop#83/OI#55	ECs1697 (Z1964)	putative ferric enterobactin transport ATP-binding protein	100%	100%	0%
S-loop#83/OI#55	ECs1698 (Z1965)	putative ABC transporter permease protein	100%	100%	0%
S-loop#83/OI#55	ECs1699 (Z1966)	putative ATP-binding protein of ABC transporter	100%	100%	0%
S-loop#83/OI#55	ECs1700 (Z1967)	hypothetical protein	100%	100%	0%
S-loop#83	ECs1701 (Z1968)	trehalase, periplasmic; treA	100%	100%	0%
S-loop#83 downstream region	ECs1702	trehalase, periplasmic; treA	100%	100%	0%
S-loop#83/OI#55 downstream region	ECs1703 (Z1969)	putative PTS system enzyme I; ycgC	100%	100%	0%
S-loop#83/OI#55 downstream region	ECs1704 (Z1970)	putative dihydroxyacetone kinase (EC 2.7.1.2); b1199	100%	100%	0%
S-loop#83/OI#55 downstream region	ECs1705 (Z1971)	putative dihydroxyacetone kinase (EC 2.7.1.2); b1200	100%	100%	0%
S-loop#85/OI#71	ECs1815 (Z6020)	NleF	100%	100%	0%
S-loop#85/OI#71	ECs1814 (Z6021)	NleH1-2 homolog of *Shigella *OspG	100%	100%	0%
Sp10	ECs1929 (Z2415)	putative integrase; b1345	100%	0%	0%
Sp10	ECs1930 (Z2414)^3^	hypothetical protein; ydaQ	100%	0%	8%
Sp10	ECs1931 (Z2413)^3^	hypothetical protein; ydaC	100%	0%	8%
Sp10	ECs1932 (Z2412)	restriction alleviation and modification enhancement; lar	100%	0%	0%
Sp10	ECs1937 (Z2404)	phage superinfection exclusion protein; sieB	100%	0%	0%
Sp10	ECs1938^4^	hypothetical protein	87%	0%	0%
Sp10	ECs1939 (Z2403)	hypothetical protein, ydfA	100%	0%	0%
S-loop#91 (in Sp10)	ECs1941 (Z2400)	putative transcriptional regulator	100%	0%	0%
S-loop#91 (in Sp10)	ECs1942 (Z2399)	regulatory protein	100%	0%	0%
S-loop#92 (in Sp10)	ECs1944 (Z2397)	hypothetical protein	100%	0%	0%
S-loop#92 (in Sp10)	ECs1947 (Z2395)	hypothetical protein	100%	0%	0%
S-loop#93 (in Sp10)	ECs1950 (Z2392)	hypothetical protein	100%	0%	0%
S-loop#93 (in Sp10)	ECs1951 (Z2391)	hypothetical protein	100%	0%	0%
S-loop#93 (in Sp10)	ECs1952 (Z2390)	hypothetical protein	100%	0%	0%
S-loop#93 (in Sp10)	ECs1953 (Z2389)	putative methyltransferase	100%	0%	0%
S-loop#93 (in Sp10)	ECs1954 (Z2387)	hypothetical protein	100%	0%	0%
S-loop#93 (in Sp10)	ECs1955 (Z2386)	hypothetical protein	100%	0%	0%
S-loop#153	ECs2989^3^	putative regulatory protein	93%	0%	0%
S-loop#153/OI#93	ECs2990 (Z3358)^5^	putative prophage repressor CI	93%	0%	0%
S-loop#153/OI#93	ECs2991 (Z3359)^5^	hypothetical protein	93%	0%	0%
S-loop#153/OI#93	ECs2992 (Z3360)^5^	putative membrane protein	93%	0%	0%
S-loop#153/OI#93	ECs2993 (Z3361)^5^	putative regulatory protein	93%	0%	0%
S-loop#153	ECs2994^3^	hypothetical protein	93%	0%	0%
S-loop#153/OI#93	ECs2995 (Z3362)^5^	putative superinfection exclusion protein	93%	0%	0%
S-loop#286/OI#172	ECs5242 (Z5878)	putative integrase	80%	100%	0%
S-loop#286/OI#172	ECs5245 (Z5881)	orf; Unknown function	80%	100%	0%
S-loop#286/OI#172	ECs5246 (Z5882)	hypothetical protein	80%	100%	0%
S-loop#286	ECs5247	hypothetical protein	80%	100%	0%
S-loop#286/OI#172	ECs5248 (Z5884)	hypothetical protein	80%	100%	0%
S-loop#286/OI#172	ECs5249 (Z5885)	putative resolvase	80%	100%	0%
S-loop#286/OI#172	ECs5250 (Z5886)	hypothetical protein	80%	100%	0%
S-loop#286/OI#172	ECs5251 (Z5887)	hypothetical protein	80%	100%	0%
S-loop#286/OI#172	ECs5252 (Z5888)	putative transcription regulator	80%	100%	0%
OI#172	Z5889	orf; Unknown function	80%	100%	0%
KI#71	b1142	orf, hypothetical protein; ymfH	0%	0%	100%
KI#71	b1147	orf, hypothetical protein; ymfL	0%	0%	100%
KI#71	b1148	orf, hypothetical protein; ymfM	0%	0%	100%
KI#71	b1152	orf, hypothetical protein; b1152	0%	0%	100%
KI#121	b2360	orf, hypothetical protein; b2360	0%	100%	100%
KI#121	b2361	orf, hypothetical protein; b2360	0%	100%	100%
KI#121	b2362	orf, hypothetical protein; b2362	0%	0%	100%
KI#121	b2363	orf, hypothetical protein; b2363	0%	0%	100%
backbone	b1201	putative sensor-type regulator; b1201	0%	100%	100%
backbone	b1202	putative adhesion and penetration protein; b1202	0%	100%	100%
backbone	ECs2126 (b1519)	putative enzyme	100%	100%	0%
backbone	ECs2127 (b1520)	orf, hypothetical protein	100%	100%	0%

^1^Divergent in lineage II strains and the lineage I strain 97701.

^2 ^Divergent in lineage I strain 97701 and 11/12 lineage II strains

^3 ^ECS1930-ECS1931 were divergent in 11/12 lineage II and 4 lineage I/II strains. They were present in the lineage I strains and one lineage II strain.

^4^ECs1938 was divergent in lineage II strains, however, it was absent in lineage I strains 63154 and E2328.

^5^Divergent in Lineage II strains and the lineage I strain LRH6.

**Table 5:** Conditions used for PCR amplification of *E. coli *FRIK 920 genomic regions

Region	Primer Sequences	Ta, °C
S-loop#14, OI#7	ECs0236: 5'ggtaataccggcagacagaacatgg3'	68
	ECs0244: 5'gtagcgcagaactccatattctcc3'	
S-loop#16, OI#8	NA	
S-loop#69, OI#45	ECs1160 (upstream): 5' ccgcctgcgatggtggttgc 3'	60.2
	ECs1252: 5' gggcgcgggtgattttgctctc 3'	
S-loop#72, OI#43/48	ECs1374: 5' aaatgagacgccagcacctatcca 3'	68
	ECs1395: 5' aagcgagtaaggcagggaggagag 3'	
S-loop#78, OI#51	ECs1575: 5'taaccacgctaccagtagccagaag 3'	68
	ECs1601: 5' gctactacctgcatcgtgccagtat 3'	
S-loop#83, OI#55	ECs1688 : 5'caccagtgcctgccagtcaatatct3'	52
	ECs1707: 5'atgtcaacgacgcctctggatatgc3'	
S-loop#85, OI#71	NA	
S-loop#91/92/93	ECs1927: 5' tgcctcccgcccaactcacg 3'	60
	ECs1958: 5' tagtcatcccccgccccacataac 3'	
S-loop#153, OI#93	NA	
S-loop#286, OI#172	ECs5240: 5' ggcacgccgatttccgacaa 3'	68
	ECs5253: 5' aagcaaccgcccccgacatc 3'	
KI#71	b1142: 5' aactggtaccgccaagactacac3'	ND
	b1156: 5' ccgatactgaagcacagcatagc3'	
KI#121	b2360: 5' taatatgcgtgccgtcagtgtgc3'	ND
	b2363: 5' ctgccagatgatccaaccgagag3'	
b1518-20 (backbone)	b1520/Z2184: 5' gttctacgctggtggaccgtatc3'	68
	b1518/Z2187: 5' agatgaagacgcagtggcgttcc3'	
